# Signaling pathway perturbation analysis for assessment of biological impact of cigarette smoke on lung cells

**DOI:** 10.1038/s41598-021-95938-z

**Published:** 2021-08-18

**Authors:** Hongyu Chen, Xi Chen, Yifei Shen, Xinxin Yin, Fangjie Liu, Lu Liu, Jie Yao, Qinjie Chu, Yaqin Wang, Hongyan Qi, Michael P. Timko, Weijia Fang, Longjiang Fan

**Affiliations:** 1 Department of Medical Oncology, First Affiliated Hospital, Zhejiang University, Hangzhou, 310058 China; 2 Institute of Crop Science, Zhejiang University, Hangzhou, 310058 China; 3 Institute of Bioinformatics, Zhejiang University, Hangzhou, 310058 China; 4 Department of Bioinformatics and Computational Biology, The University of Texas MD Anderson Cancer Center, Houston, TX 77030 USA; 5 Institute of Biotechnology, Zhejiang University, Hangzhou, 310058 China; 6 Department of Pathology and Pathophysiology, School of Medicine, Zhejiang University, Hangzhou, 310058 China; 7 Department of Biology and Public Health Sciences, University of Virginia, Charlottesville, VA 22904 USA

**Keywords:** Cell biology, Risk factors

## Abstract

Exposure to cigarette smoke (CS) results in injury to the epithelial cells of the human respiratory tract and has been implicated as a causative factor in the development of chronic obstructive pulmonary disease and lung cancers. The application of omics-scale methodologies has improved the capacity to understand cellular signaling processes underlying response to CS exposure. We report here the development of an algorithm based on quantitative assessment of transcriptomic profiles and signaling pathway perturbation analysis (SPPA) of human bronchial epithelial cells (HBEC) exposed to the toxic components present in CS. HBEC were exposed to CS of different compositions and for different durations using an ISO3308 smoking regime and the impact of exposure was monitored in 2263 signaling pathways in the cell to generate a total effect score that reflects the quantitative degree of impact of external stimuli on the cells. These findings support the conclusion that the SPPA algorithm provides an objective, systematic, sensitive means to evaluate the biological impact of exposures to CS of different compositions making a powerful comparative tool for commercial product evaluation and potentially for other known or potentially toxic environmental smoke substances.

## Introduction

Cigarette smoke (CS), which also called mainstream smoke (MS), is the leading cause of disease of the human airway worldwide, including chronic obstructive pulmonary disease (COPD), asthma and lung cancer^1^. Because of its effects on human health, smoking and smoking related diseases have attracted great concern from the public and the government (Such as FDA, PHE, etc.). Conventional cigarettes with low tar or nicotine content^2^, electronic cigarettes generating nicotine-containing vapor instead of CS^3^, and Heated Tobacco Products with purported reduced levels of combustion generated toxic chemical constituents^4^ have been developed for individuals unwilling or unable to quit smoking. Understanding the health effects of different tobacco products is extremely important for the public and requires not only the determination of the smoke constituents but also their biological effects.

Historically, studies of the adverse effects induced by CS mainly focused on examing the release of some toxic substances in CS^5^. However, CS is an extremely complex and dynamic aerosol, which contains more than 6000 identified chemical compounds^6^. So chemical composition analysis can’t fully characterize the possible harm of CS to the human body. Biological evaluations of CS to human health risk could provide more accurate approaches to reflect the actual harm and address the investigation requirements. Many in vivo inhalation studies have been conducted with rodent models to reproduce and investigate the pathogenesis of airway diseases, but such results can’t be entirely and straightforwardly extrapolated to humans due to interspecies differences^7,8^. Meanwhile, in vitro air–liquid interface (ALI) models of respiratory tract tissue have also been improved to assess the genotoxicity, mutagenicity and cellular response of tobacco smoke particulate matter. These ALI models now display similar morphology and character to human in vivo tissue which enable investigations of the toxicological effects of test substances to be conducted using human cells^9–11^. According to different cell sources, it can be divided into 2D ALI models and 3D ALI models. 3D ALI models are constructed using primary cells which differentiated over a period of time to form multi-cell populations and are more similar to human tissues, while 2D ALI models mostly use single cell type, especially cell lines. However, since the primary cells are directly derived from the human body therefore with large individual differences, which will be difficult to apply to large-scale comparative analysis. 2D ALI models are selected and used in our experiments.

With such in vitro models, a variety of cytotoxicity or genotoxicity assays have been developed to evaluate the harmful effects and potential risks of CS on human health, such as the neutral red uptake (NRU) assay^12^, lactate dehydrogenase (LDH) assay^13^, methyl thiazolyl tetrazolium (MTT)^14^ and in vitro micronucleus test (MN)^15^. Omics technologies application in toxicology evaluation has also been evolving besides cellular screening approaches. Transcriptome, which reflects the genome-wide reaction to environmental stress at gene level, has been shown to be more sensitive than the effect indicators at individual-level. The effect endpoint of gene expression responsive to environmental toxic substances must be earlier than the survival indicators and reproductive indicators of individual organisms. Fedorenkova et al.^16^ analyzed the correlation between the acute toxicity data and gene expression data at the biological individual level and showed that the sensitivity of gene expression to cadmium exceeded the acute toxicity indicators. In addition, RNA profiles were closely related to human disease states. RNA profiling of tumors, as one of biomedical applications, is used to predict tumor aggressiveness, understand the responses to the treatment and assess the risks of cell rejection^17,18^. In smoking risk studies, the transcriptomic data obtained from ALI bronchial tissue culture exposure models also provide deeper insights into the biological mechanisms perturbed by CS^19,20^. We utilized the in vitro model to examine differential expression of genes and found that although e-vapor did not cause many of the cytotoxic reactions observed in human bronchial epithelial cells, e-vapor exposure with or without added nicotine was not benign but elicits discrete transcriptomic signatures^21^. Not limited to transcriptome analysis, perturbation of metabolism in CS-exposed tissues, global gene expression profiles and protein alterations also can be combined. Ishikawa et al.^22^ learned that CS interfered with central carbon metabolism, oxidative stress and epidermal growth factor receptors that have been identified as key regulators of perturbation processes by using multi-omics.

To better demonstrate the biological risk of CS exposure with emerging omic data, powerful and effective cellular signal perturbation analysis methodology is required. At present only one algorithm, network perturbation amplitude (NPA)^23–29^, has been developed and applied in the quantification of biological toxicity of CS. Kuehn et al.^30^ analyzed the gene expression data with NPA to reveal the potential reduced risk of e-vapor. However, the NPA method was originally developed based on microarray datasets and only focused on specific signaling pathways of the pulmonary and vascular systems which is the most affected tissues exposed to smoking^29^. The annotation limitation of NPA constrains the application extended to non-lung cells. Furthermore, *t* test statistics which is required to analyze the microarray data in the NPA restricts the transplant of RNA-seq data which requires *Z*-statistics of the Wald test into the algorithm.

To provide more comprehensive understanding about the biological toxicology mechanism of CS exposure, we developed a human bronchial epithelial cell transcriptome-based signaling pathway perturbation analysis (SPPA) algorithm to take all cellular biological pathways into account to provide a measure of overall biological toxicity of CS and indicate specific responsive signal pathways in the cells. Here we provide evidence for the validity and accuracy of the algorithm by analyzing transcriptome profiling data derived from cell exposure in a smoking platform and in vitro air–liquid interface model.

## Methods and materials

### Cell culture

The human bronchial epithelial cell line BEAS-2B cells were obtained from the Shao laboratory (Institute of Medicine in Zhejiang University, Hangzhou, China). BEAS-2B cells were cultured in DMEM basic nutrient solution (Gibco, Thermo Fisher Scientific, China) supplemented with 10% fetal calf serum (FCS) (GR, Gibco, New York, US), 2 mM L-glutamine (AR, Solarbio, Beijing, China) and 100 units/ml Penicillin–Streptomycin (HyClone SV30010, Beijing, China) in a CO_2_ incubator (Thermo scientific, China) at 37 °C with 5% (v/v) CO_2_. The cells were passaged when they reached 80–90% confluence.

### Experimental cigarettes

Three types commercial cigarettes with different tar content (8 mg/cig, 12 mg/cig) were purchased for use in this study. They are called CB8, AB8, AB12 which the number means tar content. Specifically, CB8 is purchased from China, and AB8 and AB12 are purchased from an American tobacco company. The nicotine and tar content of cigarettes are also described in Supplementary Table S1. 3R4F reference cigarettes (University of Kentucky, Lexington, KY, USA) were also chosen for exposure experiments. All cigarettes were conditioned at 22 ± 1 °C and 60 ± 3% relative humidity for at least 48 h before being used in the experiments.

### Cell exposure treatment based on the in vitro air–liquid interface model

**Figure 1:**
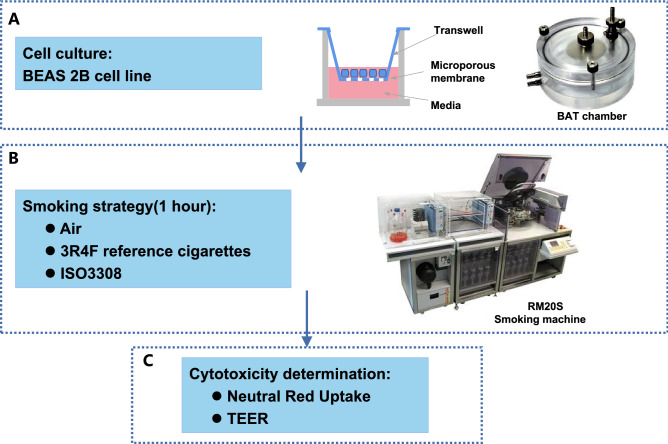
Experimental platform and workflow for assessment of biological impact of mainstream cigarette smoke. **A** BEAS 2B cells were cultured into transwells (Corning 3402) as described in the Materials& Methods. The cells were then transferred to exposure chambers for smoke exposure. **B** Cigarette smoke was generated in a Borgwaldt RM20S smoking machine under ISO3308 smoking regime and diluted cigarette smoke from a reference cigarette (3R4F) was introduced into cell exposure chanbers. **C** After treatment with the diluted cigarette smoke for 1 h, cell viability assessed by NRU assays and testing the TEER values. NRU: neutral red uptake; TEER: trans-endothelial electrical resistance. The images of Exposure chambers and Borgwaldt RM20S smoking machine are taken from Adamson et al.^52^.

For CS exposure treatment experiments, cells were seeded onto transwell inserts (Corning Incorporated, US) with 3 μm polycarbonate membrane at a density of 2.0 × 10^4^ cells/cm^2^ and thereafter an ALI was established by removing the medium from the apical surface, exposing only the basal surface of the cells to medium. Cells were further used for subsequent air and CS exposures by Borgwaldt RM20S (Borgwaldt KC GmbH, Hamburg, Germany) which is a rotary syringe smoking machine specifically designed for in vitro biological toxicity assessment of CS (Fig. 1).

The whole CS was diluted with laboratory air (Temperature 22 ± 1 °C; Humidity 60 ± 3%) to series times (10×, 30×, 100×, 300×, 500×, 1000×) to expose BEAS-2B cells maintained at the ALI in exposure chambers housed at 37 °C (Table 1). The puffing regimen was set according to the International Organization for Standardization (ISO3308:2012) with the following parameters: a 35-ml puff drawn over 2 s every 1 min period; a 5 min cycle per cigarette yielding 12 cigarettes smoked per 1 h of treatment. To avoid potential exposure to CS components in the nutrient solution due to aerosol sedimentation, a peristaltic pump was used to replace the nutrient solution at a flow rate of 3 ml/min. One-third of the treated cells after CS treatment for 1 h were washed with PBS immediately to collect cells, and the rest were cultured in CO_2_ incubator for 4 h/24 h before sampling. Cytotoxicity assays and RNA extraction were subsequently performed. The negative control groups were achieved with air-exposed cells under the same protocol with CS exposed cells.

**Table 1:** Summary of smoke exposure experiments for validation in this study.

Experiments	Cigarette type	Sample code	Tar (mg/cig)	Nicotine (mg/cig)	Dilution times	Sampling time (hour) after exposure
Optimal concentration	Reference	3R4F	9.4	0.73	1/10, 1/30, 1/100, 1/300,1/500,1/1000	0
Transcriptomic profiling: reference versus commercial cigarette	Reference	3R4F_0	9.4	0.73	1/500	0
	Reference	3R4F_4	9.4	0.73	1/500	4
	Reference	3R4F_24	9.4	0.73	1/500	24
	Commercial	CB8_0	8	0.8	1/500	0
	Commercial	CB8_4	8	0.8	1/500	4
	Commercial	CB8_24	8	0.8	1/500	24
Transcriptomic profiling: different tar/nicotine contents	Commercial	AB8_4	8	0.7	1/500	4
	Commercial	AB12_4	12	1	1/500	4
	Commercial	CB8_4	8	0.8	1/500	4

### Nicotine content determination

In order to determine the nicotine content in CS, we use cambridge filters to collect CS, and immediately extract with 20 ml absolute ethanol. Gas chromatograph was used for nicotine content detection. We tested the nicotine content of 1/500 CS and carried out six repeated experiments for each kind of cigarette.

### Trans-endothelial electrical resistance measurement

Trans-endothelial electrical resistance (TEER), an electrical parameter to assess membrane integrity and suitability of in vitro cellular barriers^31^, was used to assess cell layer integrity and toxicity of CS exposure. TEER was measured using a Millicell-ERS-2 (Millipore, Billerica, MA, US) volt-ohm-meter with a STX01 chopstick electrode (Millipore, Billerica, MA, US). Triplicate measurements were performed for each well. Three wells per experimental run were tested. Subsequently, the values were adjusted with blank control. The obtained value was multiplied by the effective membrane area in cm^2^ (1.12 cm^2^ for 12-well transwell inserts) to yield the final result in Ω.cm^2^.

### Neutral red dye uptake assay

Neutral red uptake (NRU) is the most widely used assay for the assessment of cytotoxicity in the context of tobacco product testing. Neutral red is an acidotropic stain that is taken up by lysosomes. Lysosomal membranes are damaged by cytotoxic substances so that the uptake and binding of the dye is decreased. The cell samples were incubated for 3 h with nutrient medium containing 0.5% neutral red. The dye was extracted, and the absorbance measured at 540 nm using a spectrophotometer (Synergy H1, USA). Cell viability was calculated by determining the ratio of absorbance in treatment group compared to the control group. Three wells are tested for each experiment run.

### Transcriptomic profiling of BEAS 2B cells under smoke exposure

Total RNA was isolated with the RNeasy mini kit (QIAgen, Hilden, Germany) following the manufacturer’s procedure. RNA was quantified using a NanoDrop ND1000 (NanoDrop Technologies, Wilmington, DE, USA). Samples with a RIN number greater than 8 were retained for subsequent analyses. Paired-end sequencing was then performed on an Illumina Hiseq 4000 for RNA-seq. Triplicates were made for each treatment and control, which a total of 39 samples were achieved according to the experimental design. All the sequencing data generated in this study was submitted to NCBI with accession number PRJNA637969.

FASTQC^32^ was used to generate quality control (QC) metrics for initial reads and low quality read ends were trimmed using trimmomatic^33^. The RNA-seq reads were aligned to the human genome (GRCh38) using HISAT^34^ aligner with default parameters. Differential expression genes were analyzed by edgeR package^35^. The significantly modulated genes were further analyzed for GO classification by Clusterprofiler package^36^ with adjusted *p value* < 0.05 and fold change (FC) > 2 or <  − 2 considered as statistically significance.

### SPPA algorithm overview

To objectively address quantification of impact of CS on a human cell line, and explore biological signaling pathway perturbation caused by the whole CS, we established a methodology based on genome-wide gene expression profile data and biological signaling pathway information and developed a pipeline (“AB-smoke”) implementing the algorithm by R and Perl language. The biological pathways information was downloaded from Reactome database (V70, https://www.reactome.org/) which includes 27 topics (cell cycle, disease, cell–cell communication, programmed cell death, etc.), 2263 biological pathways with a total of 12,608 reactions. In addition, we specially collected ten cancer-related pathways^37^ to detect whether there are differences in the effects of CS from different cigarettes on these pathways. The ten pathways have been identified as frequently genetically altered in cancer and changes in gene expression of these pathways are related to the occurrence and development of cancer. The approach comprises four steps, using gene expression profiles and biological pathways information as input, and the total effect scores which reflect the overall biological risk of the cigarettes and detailed quantification of the disturbance of different signaling pathways as output. The SPPA algorithm can be downloaded from https://github.com/bioinplant/SPPA.

#### Step 1: Calculation of effect score for each biological signaling pathway

**Figure 2:**
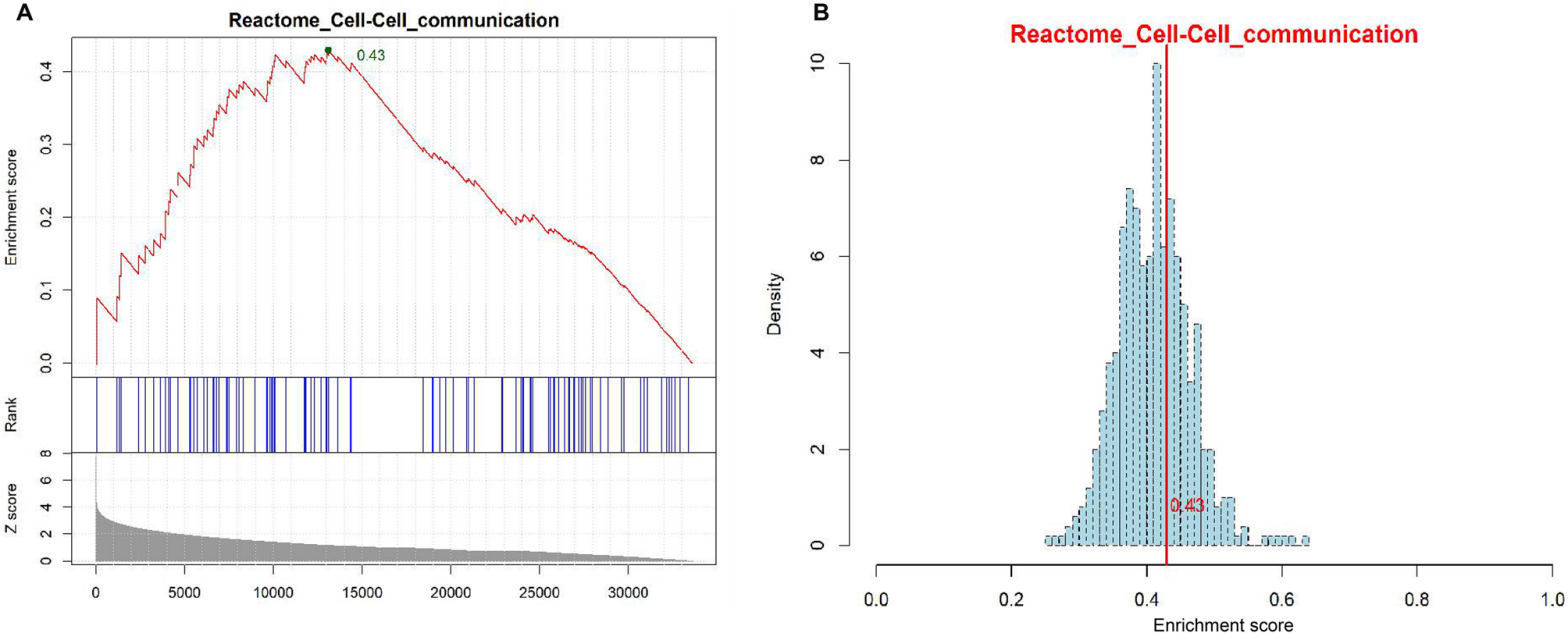
Illustration of the SPPA algorithm. **A** Shown is the estimation of the effect score (ES) of the “Cell–Cell communication” pathway. When all 112 genes are sorted according to the Z values of expression changes in the pathway, the highest enrichment score of the pathway based on running-sum statistic is calculated by finding the location of genes in the pathway. The score is 0.429, which is as effect score of smoke on this pathway. **B** Estimation of significance of effect score. 500 random simulations were performed to calculate whether the ES (0.429) of the example pathway reached a significant level. The estimation of *P* value is 0.365.

In order to study the overall effect of smoke on cells, first we need to evaluate the effect of smoke on each of the biological signaling pathways of the cell through transcriptomic data. However, if you only focus on differentially expressed genes, there may be some disadvantages, and you fail to fully use the information of the transcriptome, cellular processes often affect a coherent set of genes. A 20% increase of all genes involved in a biological pathway may significantly alter the expression flux of that pathway, and may be more important than a ten-fold increase in individual genes in the biological pathway^38^. Therefore, for the analysis of biological signal pathways, we borrowed the gene set enrichment analysis (GSEA) to comprehensively consider all gene expression transformations. First, we get ranked list L of all the genes on the data based on the difference of their expression levels between the smoke versus air. We sort all genes based on the absolute fold change value of the expression change (*Z* value). Then, for each gene set S: find the location of each gene in S within L. Generate enrichment score for S based on running-sum statistic. For each of the designated biological pathways, the score is calculated by walking around the list of all genes that have been sorted. When we encounter a gene in the list, it will increase a running-sum statistic, otherwise it will be decreased (for example see Fig. 2A). The specific addition and subtraction algorithms learn from the GSEA algorithm. Here we use the maximum score obtained during the calculation as effect score (ES) of smoke on a biological pathway that reflects the degree of disturbance. If the genes in the specified pathway randomly distributed in the sorted gene list or the whole genes in the pathway does not show differential expression, the maximum can always find at the bottom; if the genes in the specified pathway distributed at the top of the sorted gene list, the effect score will be found at the top of the sorted gene list.

$${P}_{hit}\left(S,i\right)=\sum_{\begin{array}{c}{g}_{j}\in S\\ J\le l\end{array}}\frac{|{r}_{j}|}{{N}_{r}}$$, where $${N}_{R }= \sum_{{g}_{j}\in S}|{r}_{j}|{P}_{hit}\left(S,i\right)=\sum_{\begin{array}{c}{g}_{j}\in S\\ J\le l\end{array}}\frac{|{r}_{j}|}{{N}_{r}}$$

$$ P_{{miss}} \left( {S,i} \right) = \sum\nolimits_{{\begin{array}{*{20}c}    {g_{j}  \in S}  \\    {J \le l}  \\   \end{array} }} {\frac{1}{{(N - N_{H} )}}}  .$$

The ES is the maximum deviation from zero of ${P}_{hit}\left(S,i\right)-{P}_{miss}$. For a randomly distributed S, ES(S) will be relatively small, but if it is concentrated at the top or bottom of the list, or otherwise non-randomly distributed, then ES will be correspondingly high.

We estimate the significance of an observed ES by comparing it with the set of scores ES null computed with randomly assigned phenotypes (Fig. 2B):

1. Randomly assign the original phenotype labels to samples, reorder genes, and re-compute ES(S).
2. Repeat step 1 for 500 permutations and create a histogram of the corresponding enrichment scores ES null.
3. Estimate nominal *P* value for S from ES null by using the positive or negative portion of the distribution corresponding to the sign of the observed ES(S). And calculate the normalized *Z*-score of each gene set (Fig. 2B provides an example).

Taking the “cell–cell communication” pathway in the Reactome database as an example to calculate its effect score. The positions of 112 genes contained in the pathway is found by traversing from left to right the gene list that has been sorted according to the expression change folds (Fig. 2A). When we encounter a gene in the list, it will increase a running-sum statistic, otherwise it will decrease. The highest enrichment score of cell–cell communication pathway is calculated by finding the location of gene, and finally its effect score is 0.45 (Fig. 2A). We further randomly select the same number of genes (112 genes) in the ranked list to calculate the effect score. Through 500 random simulation calculations, we constitute a zero distribution for the pathway to estimate the statistical significance of the effect score of the pathway. Based on the obtained distribution, we calculate a standardized *Z*-score (0.429) based on the effort score. For cell–cell communication pathway, its *P*-value is 0.365, which does not meet the significance requirement (< 0.05) (Fig. 2B) and will not be included in the further estimation of total effect score.

#### Step 2: Weight effect score by pathway gene number

Different pathways contain different numbers of genes and some pathways contain only a few genes or have not been thoroughly defined. For example, AKT2 pathway only contains four genes while the GPCR pathway has 1220 genes. As a consequence, the contribution of different pathways to the overall effect will vary. Therefore, to define the impact of each pathway, we need to consider the influence of the number of genes present. We further calculated ES from the log-transformed value of the number of genes (Ng) in a particular pathway and the *Z*-score (Z) of ES normalized by the impact score based on the permutation results in the first step.

$$ ES = {\text{log}}(N{\text{g}}) \times Z $$

#### Step 3: Calculation of effect score for main functional categories

In the Reactome database, 2263 biological signal pathways are divided into 27 functional categories, including cell cycle, hemostasis, developmental biology, etc. Each category contains a different numbers of sub-channels and presents a clear hierarchical structure. In order to give the degree of influence of smoke exposure on cells in each large category, also to standardize each category can be used for comparison. We use the significant differential gene pathways found in the first step and the weighted effect scores calculated in the second step to calculate the normalized effect score (NES) for the main functional categories. The NES is the product of the average number of the ES of the subcategories and the number of the subcategories by log2 transformation.

#### Step 4: Calculation of total effect score

Based on the NES of the main functional categories calculated in the third step, we therefore can calculate the total effect score (TES) of the cigarette. This score is the sum of effect scores for all major functional categories.

## Results

### Determination of optimal concentration for smoke exposure

**Figure 3:**
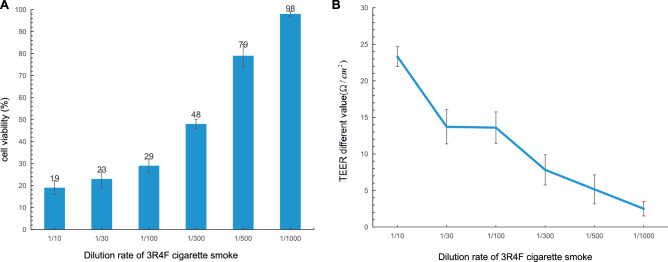
Different endpoints of assessing the effect of cigarette smoke dilution rates. **A** Cell viability as determined by neutral red uptake (NRU) assay under six different dilutions of 3R4F reference cigarette smoke. **B** Changes of TEER following treatment with different dilutions of 3R4F smoke. C. Boxplot of nicotine content in different kind of cigarette smoke under 500 times dilution. The mean value is shown by the horizontal line in the middle. **A t-test significance at *p* < 0.05 using the average nicotine content of other three cigarettes (3R4F, AB8, CB8) as comparator.

To test the SPPA methodology, a pilot experiment was performed using the 3R4F reference cigarette to define the optimal time of exposure and concentration to elicit a reproducible response in the HBECs relative to cell death. The experiment schematic, including the smoking strategy, exposure instrument, cytotoxicity assay, is shown in Fig. 1. Whole CS from 3R4F cigarettes was diluted using the dilution syringes on the Borgwaldt RM20S smoking machine to achieve a series aerosol doses (i.e., 1/10, 1/30, 1/100, 1/300, 1/500, 1/1000 of whole CS) (Table 1). Following exposure for 1 h, NRU and TEER analyses were immediately carried out to assess the cell viability (Fig. 3). Both analyses showed similar tends and based on these results the 1/500 dilution of the whole CS, yielding about 80% viability under NRU analysis (Fig. 3A) and a TEER value of 5.2 Ω.cm^2^ (Fig. 3B) was deemed to be the optimal dilution factor for in vitro exposure. Cells exposed to air which were left in the insert well and incubated in the incubator were compared to 1/1000 concentration exposure. No significant difference of NRU or TEER was observed. In brief, the results suggested that the biological impact on the cells of the cigarette whole smoke was affected in a dose dependant manner, and 1/500 was the optimal concentration of the whole smoke which was used for next analyses in this study. At the same time, we measured the nicotine content of diluted cigarette smoke (1/500) by using cambridge filters (as shown in Fig. 3C). The nicotine content of AB12 is significantly higher than that of other three cigarettes.

### Transcriptomic profiling of bronchial epithelial cells under smoke exposure

To apply the SPPA algorithm on signal pathway perturbation assessment by 1/500 CS, we first applied whole transcriptome sequencing for all samples from different exposure strategies (Table 1). Triplicates were made for each treatment and control and average 7 Gb of paired-end RNA-seq data for each sample were generated. After trimming of reads, over 95% reads were successfully mapped to the human reference genome (GRCh38).

**Figure 4:**
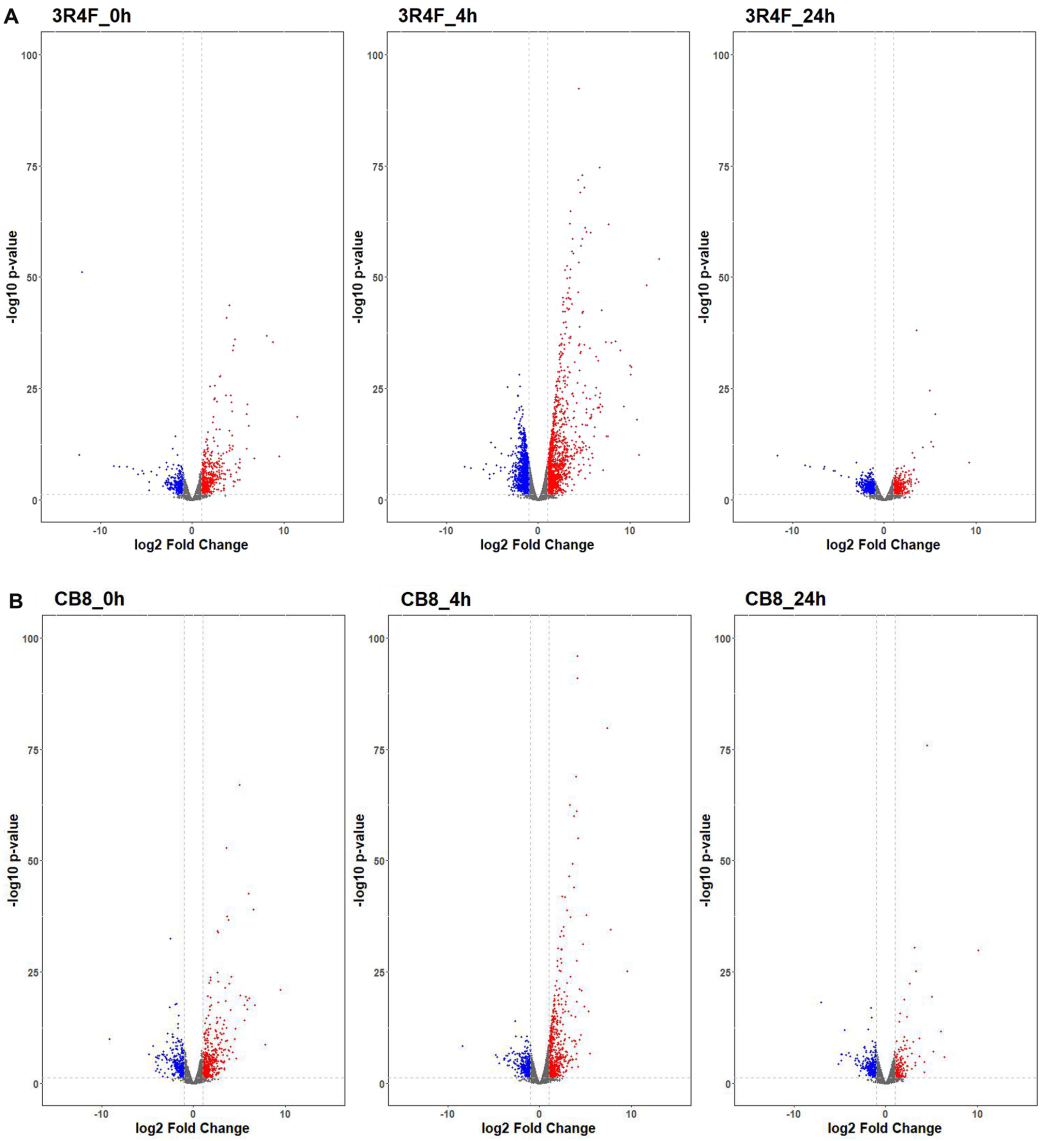
Volcano plots for mainstream smoke (MS) exposure generated by reference and commercial cigarettes. **A** Exposure to 1/500 MS from 3R4F cigarettes for 1 h, compared with the air control, the number of differential genes changes at three time points, 3R4F_0h: recovery 0 h after exposure to 1/500 MS from 3R4F cigarettes for 1 h; 3R4F_4h: recovery 4 h after exposure to 1/500 MS from 3R4F cigarettes for 1 h; 3R4F_24h: recovery 24 h after exposure to 1/500 MS from 3R4F cigarettes for 1 h. **B** Exposure to 1/500 MS from CB8 cigarettes for 1 h, compared with the air control, the number of differential genes changes at three time points. CB8_0h: recovery 0 h after exposure to 1/500 MS from CB8 cigarettes for 1 h; CB8_4h: recovery 4 h after exposure to 1/500 MS from CB8 cigarettes for 1 h; CB8_24h: recovery 24 h after exposure to 1/500 MS from CB8 cigarettes for 1 h. The X-axis displays Log2 of the fold change (FC), and the Y-axis displays -Log10 of the adjusted p-value (pFDR). The dashed horizontal lines represent the 0.05 pFDR threshold. The vertical dashed line shows + 2 and − 2 times the change threshold. Blue dots represent significant RNA features at pFDR < 0.05 and FC <  − 2, and red dots represent significant RNA features at pFDR < 0.05 and FC >  + 2.

To further evaluate the effect of 1/500 CS exposure, transcriptional activity was investigated via DEG (differentially expressed genes) analysis. We first compared the gene expression in BEAS-2B cells exposed to CS generated by reference cigarette 3R4F smoking using air-treated samples as our control and an initial 1 h exposure. A total of 720 genes were significantly (pFDR < 0.05, |FC|> 2) differentially expressed (Fig. 4 and Supplementary Table S1). Of these genes, 447 were up-regulated, suggesting that many cellular processes were stimulated following the toxic insult. The GO analysis of all DEGs at this time point also indicated that several pathways related to stimulus response are affected among which negative regulation of transferase activity, response to external stimulus and epithelial cell proliferation were significantly enriched (Supplementary Figure S1A). Meanwhile protein kinase-related pathways were activated, suggesting that the cells attempt to recover from the CS exposure.

Expression patterns at different time points could give us hints about the temporal and spatial patterns of the potential harm of CS before we quantify them by SPPA algorithm. At 4 h, the majority (1418/3082) of DEGs of 3R4F_4 were up-regulated and 2557 genes were newly activated at this time point (Fig. 4). These significantly enhances in expression pointing to a fierce response of cellular activity. The response categories were overrepresented with protein localization movements which mainly contribute to stress stimulus and possibly enable DNA damage repair or precede apoptosis. In 3R4F_24 samples, most DEGs (2761, 89.6%) in 3R4F_4 returned to normal expression level at this time point, while 278 genes expression were still significantly differential expression (Fig. 4A, Supplementary Table S1). In total, 599 genes were significantly up- or down-regulated in 3R4F_24, most of which clustered in pathways involved in ribosome and rRNA metabolism. Only 110 genes kept expressing through the whole treatment procedure in 3R4F exposure experiments. This is not surprising rapid changes in gene expression patterns would be expected in cells undergoing the effects of CS exposure whereas during the 4 h and 24 h periods post-exposure readjustment and renewal of cellular activity likely initiate and persist. This persistence has already been noted in previous studies from our group and others^19,^^39^. The similar expression patterns could also be noted from CB8 samples (Fig. 4B, Supplementary Figure S2, Supplementary Table S1). Although the cells were subjected to disturbances in CB8 treatment, the relevant pathways were different, but after 1 h of whole cigarette smoke treatment, the cells showed a sustained injury response. It is worth mentioning that the most abundant GO terms of CB8_0 refers to perturbation of negative regulation of MAPK cascade, response to heat, and stress response, with significant q-values ranging from 6.13e^-5^ to 0.03 (Supplementary Figure S2). MAPK signaling pathways plays a key role in the regulation of many cellular processes including proliferation, differentiation, the stress response, motility, growth, differentiation, survival, and death. Abnormal MAPK signaling may contribute to increased or uncontrolled cell proliferation and/or resistance to apoptosis. The KEGG enrichment analysis supported the findings above (Supplementary Figure S3). Even though there were fewer enriched pathways, MAPK signaling pathway occurred in both CB8_0 and CB8_4.

The DEGs of different commercial cigarettes CB8 (887), AB8 (671) and AB12 (2560) were also analyzed to further confirm that the 1/500 CS exposure for 1 h and recovery for 4 h was able to trigger adequate cells response (Supplementary Table S1 and Figure S4). The GO terms ID found in common between CB8 and AB8 relates to negative regulation of kinase activity and negative regulation of protein kinase activity. However, the GO enrichment of AB12 treatment mainly focused on the mitochondrial-related pathways (Supplementary Figure S5). The studies confirmed that the cells quickly responded to the 1/500 exposure of CS and the effects persisted for at least 4 h.

### Application of SPPA algorithm for assessment of biological impact: reference versus commercial cigarettes

**Figure 5:**
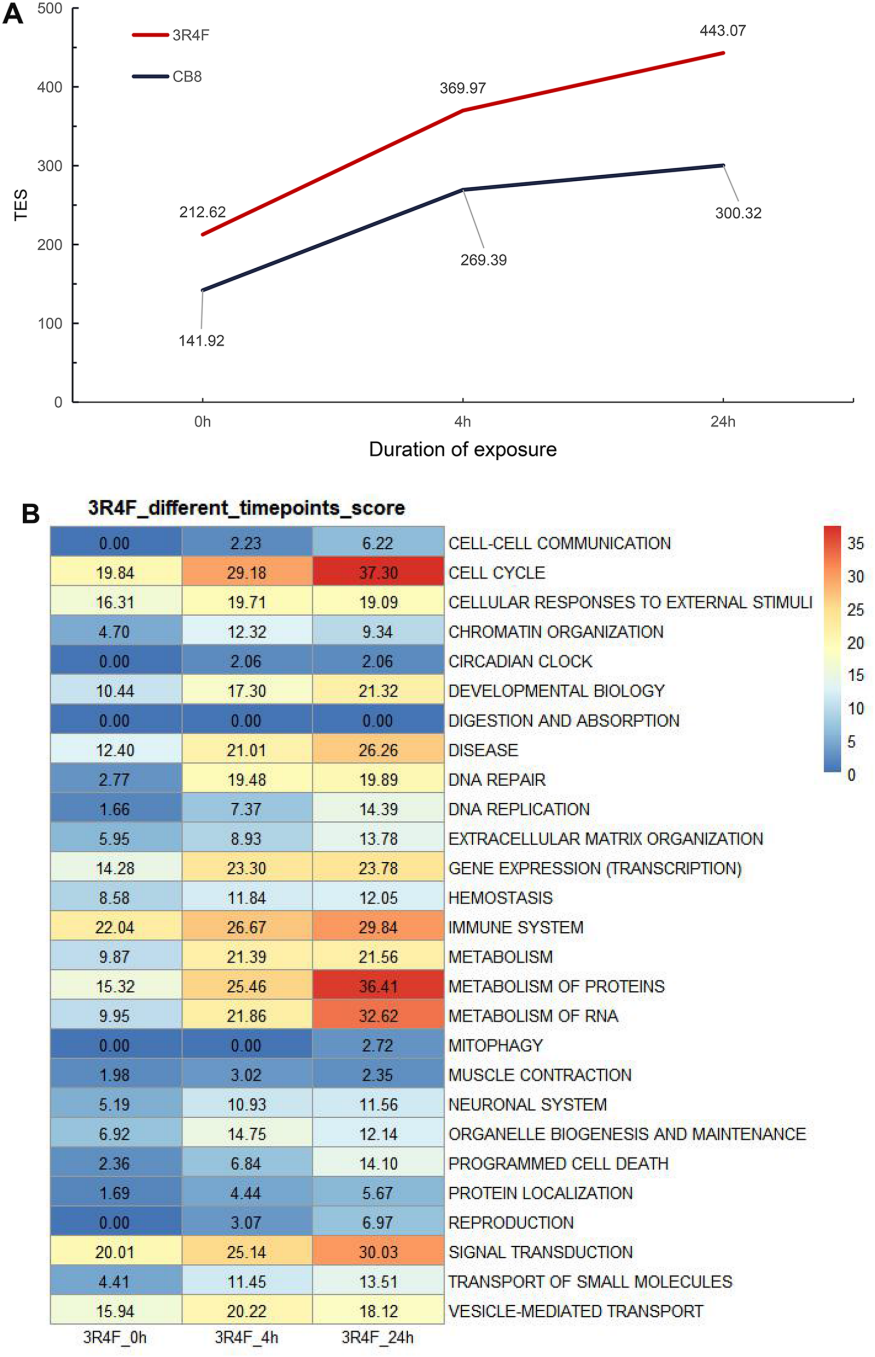

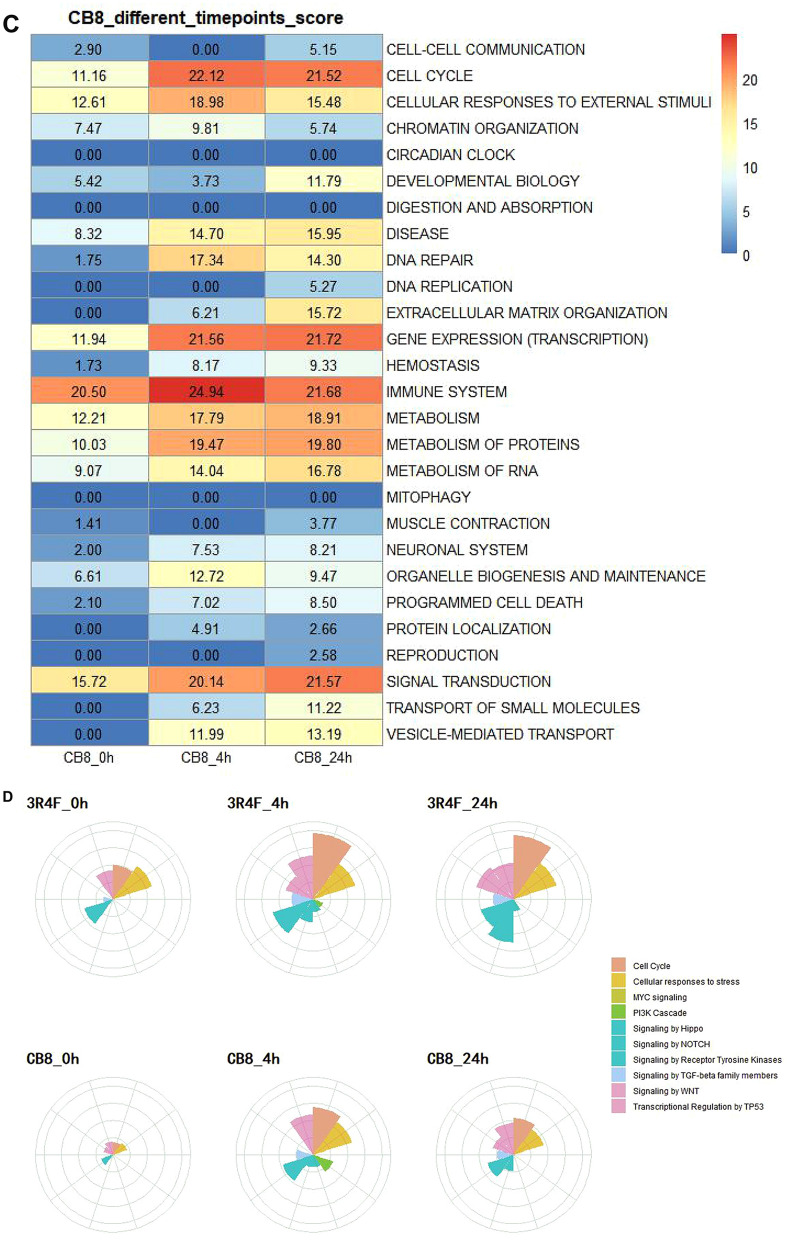
Application of the SPPA algorithm in comparisons of the biological impact of MS from reference versus commercial cigarettes. **A** The total effect scores (TES) for three time points after reference 3R4F and commercial CB8 smoke exposure using SPPA algorithm. **B**, **C** The degree of effect of 3R4F (**B**) and CB8 (**C**) smoke exposure on 27 cell-related topics at different time points. The redder the color, the higher the effect score. **D** The Z-score values of ten cancer-related pathways at different time points under 3R4F and CB8 treatment using SPPA algorithm. The larger the sector area, the larger the Z-score value, indicating the greater the perturbation to the pathway.

To access the potential application performance of the SPPA algorithm on health risk evaluation of conventional tobacco products, we applied the method using two CS treatment experiments (Table 1). The first experiment is the scoring comparison between reference cigarettes 3R4F and commercial cigarettes (CB8) (Fig. 5A). This experiment serves to evaluate the robustness of the SPPA algorithm in time-manner toxicology measurement. The second one is to compare two kind of commercial cigarettes, which contain different tar contents (8 and 12 mg/cigarette, means CB8 vs AB8 vs AB12).

Exposure to 1/500 CS from 3R4F cigarettes for 1 h, nine signaling pathways category, comprising of 282 reactions, were triggered and its TES was scored as 212.62 (Fig. 5A, B). We compared to the analysis of the transcriptomic profiles (Fig. 3), the pathway categories were also mainly cellular response to external stimulus. Overall TES increased across the recovery period, from 369.97 (3R4F_4) to 443.07 (3R4F_24), consistent with the trend in CB8 samples (Fig. 5C). The top 5 pathways in 3R4F_0, immune system, signal transduction, cell cycle, cellular responses to external stimuli and vesicle-mediated transportation, were the largest contribution to TES, and most of them showed the highest amplitude of increase at all time points. The scorings of metabolism of protein and metabolism of RNA were sharply increased to 2.5 times from 0 h (15.32, 9.95) to 24 h (36.41, 32.42) and ranked as second and third of all pathways. As expected, immune system was also computed as the highest activated pathway in CB8_0 to show the cell quick response to the CS exposure. There were only immune system and signal transduction scored over 15.00 in CB8_0, and the score of immune system has declined from 24.94 (CB_4) to 21.68 (CB_24) at the end of the experiment. It indicated that the CB8 might have less harm than 3R4F, paralleling the smaller TES of CB8 gained from the SPPA. While the number of DEGs in the cells largely decreased after 24 h of recovery, the top scores still hit cell cycle pathway in both 3R4F_24 and CB8_24. Interestingly, the score of immune system was even higher than cell cycle in CB8_24. Overall, the finding supported that the cell cycle is the primary process during perturbation reported by researchers^40^.

We narrowed down the gene sets to ten main cancer related pathways^40^, including cell cycle, signaling by Hippo, signaling by NOTCH, signaling by WNT, transcriptional regulation by TP53, MYC signaling, signaling by TGF-beta family members, cellular responses to stress, signaling by receptor tyrosine Kinases, PI3K cascade, and presented the *Z*-score generated by SPPA algorithm for these ten main pathways (Fig. 5D). After 3R4F treatment, the Z-score value of multiple pathways increased at 4 h and 24 h compared to 0 h, and at the same time, there was no significant weakening trend at 24 h. In the CB8 treatment, the Z-score of multiple pathways reached a peak at 4 h, and decreased to a certain extent at 24 h. However, its Z-scores are always lower than the value of corresponding 3R4F time point.

In this experiment, the assessed biological impact of CS on BEAS-2B cells displayed the expected time-dependent pattern of response. Moreover, the SPPA methodology also distinguish the potential difference in biological activity between reference cigarettes and commercial ones.

### Application of SPPA algorithm for assessment of biological impact: cigarettes with different contents of tar/nicotine

**Figure 6:**
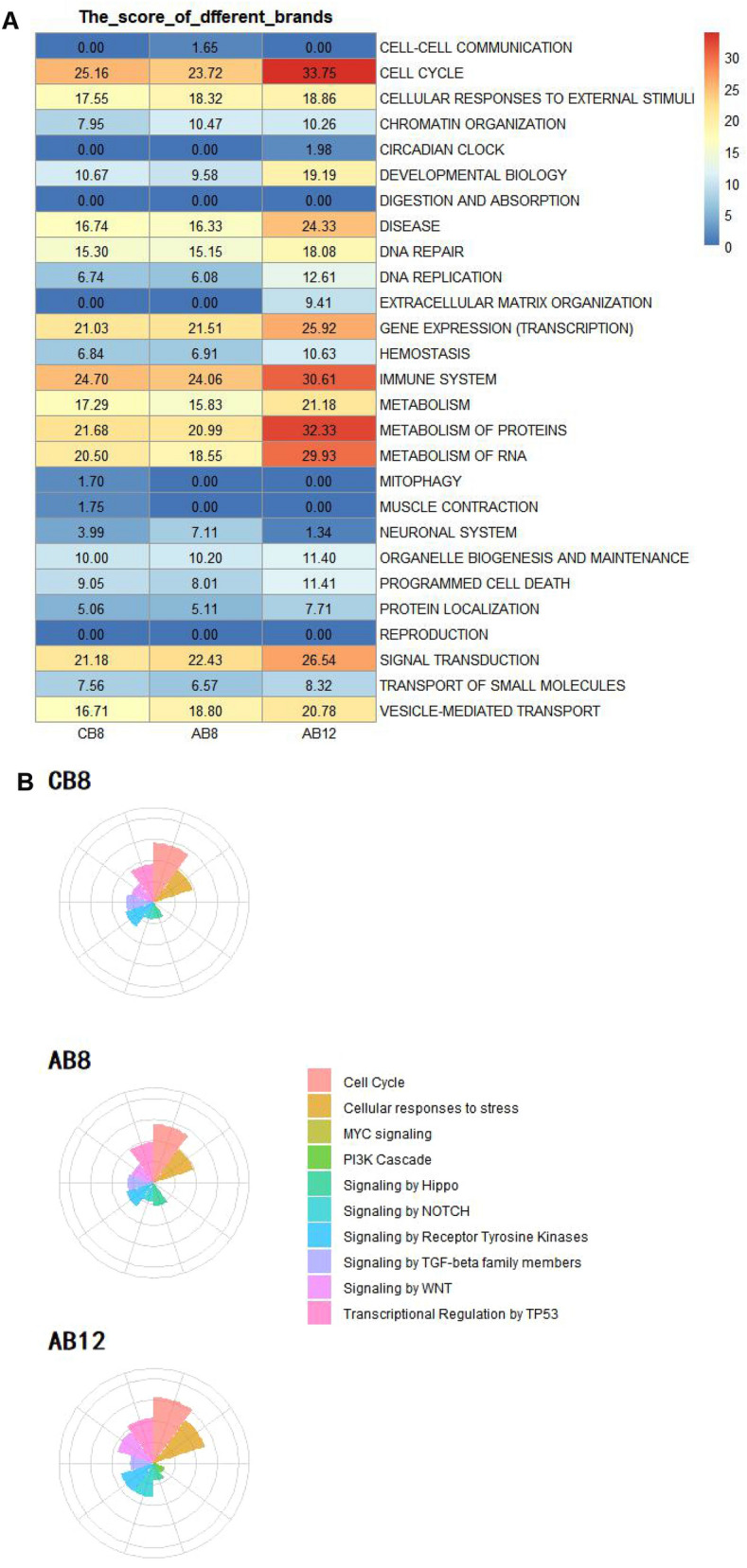
Application of SPPA algorithm in comparisons of the biological impact of MS from cigarettes with different contents of tar/nicotine. **A** The degree of effect of CB8, AB8 and AB12 smoke exposure on 27 cell-related topics at different time points. The redder the color, the higher the effect score. **B** The Z-score values of ten cancer-related pathways under CB8, AB8 and AB12 treatment using SPPA algorithm. The larger the sector area, the larger the Z-score value, indicating the greater the perturbation to the pathway. Two commercial cigarettes with different labeled tar contents, CB8 (8 mg) and AB8/AB12 (8 and 12 mg), were produced by two different tobacco companies.

In a second experiment to test the SPPA algorithm, three commercial cigarettes with different labeled tar contents were evaluated (Table 1). CB8 (8 mg per cigarette), AB8 (8 mg), and AB12 (12 mg) are manufactured by two different tobacco companies (e.g., CB and AB, respectively). Higher tar cigarettes, when puffed the same as lower tar cigarettes, should provide a larger cellular response. Comparison of AB8 and AB12 can detect the accuracy of the SPPA algorithm, while CB8 and AB8 with the same tar content shows the sensitivity of the SPPA algorithm. Consistent with this, the TES of AB12 (386.57) was much higher than that of AB8 (287.38). We next looked at the network scores to identify the significantly perturbated biological processes by AB12 or AB8 smoke. Cell cycle, and immune systems were still the primary contributors to the TES scores in both samples (Fig. 6A) while the amplitude of perturbations in these aspects under the treatment of AB12 (31.75, 30.61) was significantly higher than AB8 (23.72, 18.55). The increased scores were in agreement with previously published findings^21^ for airway epithelial cells exposed to CS. Interestingly, we observed extracellular matrix organization be specifically scored in AB12 (Fig. 6A). Although the magnitude and statistical significance of these pathway perturbations vary widely between different tar contents, the response patterns between AB8 and CB8 are similar (Fig. 6A). The TES of AB8 (287.38) was slightly lower than CB8 (289.14), the overall consistency of scores of cell cycle and immune systems in comparison between AB8 and CB8 was also observed. The two major contributors, signal transduction and vesicle-mediated transport, showed higher signal intensity in AB8 than CB8 (Fig. 6A). In agreement with these results, the similar perturbation amplitude of 10 cancer-related pathways was observed between CB8 and AB8. The most pronounced effects seen in higher tar content samples AB12 compared with CB8 or AB8 were the signaling by WNT and signaling by NOTCH.

In this experiment, the scoring methods produced global TES scores that were consistent with our expectations, particularly the separation between 8 mg tar cigarette and 12 mg tar CS treatment. The degree of influence of CS on cells has a positive correlation with tar content. For AB8 and CB8 containing the same tar content, the overall effect is similar, even though there are slight differences in some specific cellular responses. The effects of smoke on cells are mainly concentrated in the cell cycle, immune system, signal transduction and metabolism-related pathway. Moreover, the hierarchical structure of the pathways was used to gain a mechanistic understanding of the biological impact of the CS exposure by the SPPA.

In summary, based on the transcriptomic data, we provide robust SPPA algorithm which is accurate and sensitive in its ability to evaluate the cellular responses to differences in cigarette smoke exposure. By considering all expressed genes as input, our SPPA methodology could comprehensively offer perturbation information for all signaling pathways and accurately score each affected disease-related process to provide insight into the pathology and the potential of smoking-induced diseases.

## Discussion

As the increasing needs of the biological risk assessment of smoking cigarettes and the fast developing of sequencing technologies, cigarette health harm evaluation has been improved to the level of omics. In particular, transcriptome analysis has been applied to evaluate the effects of aerosols produced by different electronic cigarettes, tobacco heating products and CS on in vitro cell models^19,^^39^. However, there is still no appropriate algorithm to calculate the cell response at cellular pathway level based on omics data. The SPPA algorithm developed in this study focus on comprehensive signaling pathways and provides an effective quantitative tool by integrating transcriptomic data to accurately assess biological risk of CS. Notably, we described a unified and coherent framework for scoring individual biological signaling pathways to reflect systematic activated processes which potentially cause diseases. The results demonstrated here provide a significant extension to conventional chemical composition examination methods^41–44^, and in vitro cellular toxicity endpoints analysis^19,^^39^^,^^45,46^. The first application of the methodology on analyzing the affection of reference cigarettes provides solid evidences that the SPPA algorithm could successfully assess the biological perturbation of cigarette smoke and evaluate the time-manner effect, which results are in agreement with previous reports^21^. The further validation of the methodology illustrates the envisioned utility to comparatively assess the cigarettes with the same amount of tar which cannot be distinguished by conventional methods. Therefore, the SPPA algorithm integrating RNA-seq data could enable the accurate health risk assessment of the different types of tobacco products, including combustibles, e-vapor generating, and heat-non-burn devices, and could also be used by extension to examine other forms of environmental contaminants or toxic compounds in the air.

The SPPA algorithm is based on the RNA-seq data and ranked the all gene expression based on expression for downstream calculation facilitating the method more comprehensive and informatic. We could score all 2263 signaling pathways and abstract any interested ones by tailoring the gene sets and ascribing weights to specific pathways. In other words, compared with previous assessments based on microarray data^19^, our algorithm provides more possibilities for various researchers focus on specific topics. The envision utility is the investigation and diagnosis of disease state, the prediction of future disease onset by exposed to environmental toxic substances, such as COPD^47^. For example, all these genes for ten major cancer-related pathways^37^ could be further assigned to related processes and mechanisms that describe a coherent biological response, and could be predicted from a given experiment, further verified through additional experimental investigation. For example, we found that AB12 has a stronger effect on WNT and NOTCH signaling pathways, and this conclusion is consistent with the conclusion of the previous article^48,49^. Similarly, the methodology could also provide novel insight into the molecular mechanism of diseases and understanding the disease state by well-controlled experiments with samples from specific patients or treatments, such as healthy lung cells or tissues exposed to various inhale exposures.

In the first experiment that we considered for the SPPA validation, reference cigarette 3R4F was introduced in the system. 3R4F reference cigarette is a ‘US style’ blended product (University of Kentucky), The tar content is 9.4 mg/cig and the nicotine content is 0.73 mg/cig^50^. It is widely used to evaluate the hazard comparison of commercial cigarettes. By collecting the scores of hierarchically structured biological pathways which disturbed by 3R4F exposure, we were able to place the results into the comparison of interested tobacco products in the future application. CB8 and AB8/AB12 which utilized in the second validation experiment are commercial brands likely made with different composition of tobacco leaves. However, the TES values given by our SPPA algorithm show the same toxic levels of CB8 and AB8 (Fig. 6). Even though, it cannot be simply concluded that the health risk is linear correlated with tar content.

The application of our quantitative approach for assessing biological impact to four distinct cigarette treatments found that the results were consistent with expected responses. The cell cycle, immune systems, signal transduction, metabolism of proteins and metabolism of RNAs were the main contributors to the TES scores of all four treatments, while the contribution of the regulation of proliferation signal pathway was negligible across all time points and all treatments, which is consistent to the studies reported by Kuehn et al^30^. The activation of cancer-related signaling pathways also indicates that the composition of CS is a key factor in determining potential biohazards. This is consistent with prior studies showing that there is a dose–effect on the response of human cells^51^. Together, these experiments support our conclusion that SPPA methodology is capable of providing an accurate assessment of the biological effects of CS and its future in the evaluation of exposure to other air-borne toxic substances.

In conclusion, the SPPA scoring algorithm presented here could quantitatively assess the disturbance of CS from different cigarettes to cell biological pathways. If combined with the chemical composition analysis, cellular toxicology measurement and histology examination, it will provide more comprehensive information about the potential risks of cigarette smoke. In addition, our results highlight the features of our method as accurate and sensitive. We envision that our algorithm could have practical utility not only for systematic comparison of human health risk of various air-borne environmental toxic substance, but also for prediction of disease mechanism.

## Supplementary Information

Supplementary Information 1.

Supplementary Information 2.

## Data Availability

The RNAseq data are publicly available in Sequence Read Archive database, accession number PRJNA637969.

## Code availability

The SPPA algorithm can be downloaded from https://github.com/bioinplant/SPPA.
