# Supplementary material for: Signaling pathway perturbation analysis for assessment of biological impact of cigarette smoke on lung cells
Source: Sci Rep. 2021 Aug 18;11:16715. doi: 10.1038/s41598-021-95938-z (PMC8373939; doi:10.1038/s41598-021-95938-z)
Supplement: Supplementary file 1 — Supplementary Information 1. [file 41598_2021_95938_MOESM1_ESM.docx]

Signaling Pathway Perturbation Analysis for Assessment of Biological Impact of Cigarette Smoke on Lung Cells

Hongyu Chen^1,2,#^, Xi Chen^1,3,#^, Yifei Shen^4,#^, Xinxin Yin^1^, Fangjie Liu^3^, Lu Liu^1^, Jie Yao^3^, Qinjie Chu^3^, Yaqin Wang^5^, Hongyan Qi^6^, Michael P. Timko^7^, Weijia Fang^2,*^, Longjiang Fan^1-3,*^

*^1^ Institute of Crop Science, Zhejiang University, Hangzhou 310058, China*

*^2^ Department of Medical Oncology, First Affiliated Hospital, Zhejiang University, Hangzhou 310058, China*

*^3^ Institute of Bioinformatics, Zhejiang University, Hangzhou 310058, China*

*^4^ Department of Bioinformatics and Computational Biology, The University of Texas MD Anderson Cancer Center, Houston, TX 77030, U.S.A.*

*^5^ Institute of Biotechnology, Zhejiang University, Hangzhou 310058, China*

*^6^ Department of Pathology and Pathophysiology, School of Medicine, Zhejiang University, Hangzhou 310058, China*

*^7^ Department of Biology & Public Health Sciences, University of Virginia, Charlottesville, VA, 22904, U.S.A.*

**E-mail:** [fanlj@zju.edu.cn (Fan LJ),](mailto:fanlj@zju.edu.cn,) [weijiafang@zju.edu.cn](mailto:weijiafang@zju.edu.cn) (Fang WJ)

^#^ Hongyu Chen, Xi Chen and Yifei Shen contributed equally to this work.

**Table S1 Summary of DEG under different smoke exposure experiments in this study.**

| **Experiments** | **Cigarette Type** | **Samples code** | **Tar (mg/cig)** | **Nicotine (mg/cig)** | **Up** | **Down** | **Total** |
| --- | --- | --- | --- | --- | --- | --- | --- |
| Transcriptomic profiling: reference vs. Commercial cigarette | Reference | 3R4F_0 | 9.4 | 0.73 | 447 | 273 | 720 |
|  | Reference | 3R4F_4 | 9.4 | 0.73 | 1418 | 1664 | 3082 |
|  | Reference | 3R4F_24 | 9.4 | 0.73 | 299 | 300 | 599 |
|  | Commercial | CB8_0 | 8 | 0.8 | 412 | 294 | 706 |
|  | Commercial | CB8_4 | 8 | 0.8 | 379 | 563 | 942 |
|  | Commercial | CB8_24 | 8 | 0.8 | 248 | 109 | 357 |
| Transcriptomic profiling: different tar/nicotine | Commercial | AB8_4 | 8 | 0.7 | 424 | 247 | 671 |
|  | Commercial | AB12_4 | 12 | 1 | 1321 | 1239 | 2560 |
|  | Commercial | CB8_4 | 8 | 0.8 | 474 | 413 | 887 |
