# Supplementary material for: Signaling pathway perturbation analysis for assessment of biological impact of cigarette smoke on lung cells
Source: Sci Rep. 2021 Aug 18;11:16715. doi: 10.1038/s41598-021-95938-z (PMC8373939; doi:10.1038/s41598-021-95938-z)
Supplement: Supplementary file 2 — Supplementary Information 2. [file 41598_2021_95938_MOESM2_ESM.pdf]

Signaling Pathway Perturbation Analysis for Assessment of Biological Impact of Cigarette Smoke on Lung Cells

Hongyu Chen[1,2,#], Xi Chen[1,3,#], Yifei Shen[4,#], Xinxin Yin[1], Fangjie Liu[3], Lu Liu[1], Jie Yao[3], Qinjie Chu[3], Yaqin Wang[5], Hongyan Qi[6], Michael P. Timko[7], Weijia Fang[2,*], Longjiang Fan[1-3,*]

[1] *Institute of Crop Science, Zhejiang University, Hangzhou 310058, China*

[2] *Department of Medical Oncology, First Affiliated Hospital, Zhejiang University, Hangzhou 310058, China*

[3] *Institute of Bioinformatics, Zhejiang University, Hangzhou 310058, China*

[4] *Department of Bioinformatics and Computational Biology, The University of Texas MD Anderson Cancer Center, Houston, TX 77030, U.S.A.*

[5] *Institute of Biotechnology, Zhejiang University, Hangzhou 310058, China*

[6] *Department of Pathology and Pathophysiology, School of Medicine, Zhejiang University, Hangzhou 310058, China*

[7] *Department of Biology & Public Health Sciences, University of Virginia, Charlottesville, VA, 22904, U.S.A.*

E-mail: fanlj@zju.edu.cn (Fan LJ), weijiafang@zju.edu.cn (Fang WJ)

[#] Hongyu Chen, Xi Chen and Yifei Shen contributed equally to this work.

A

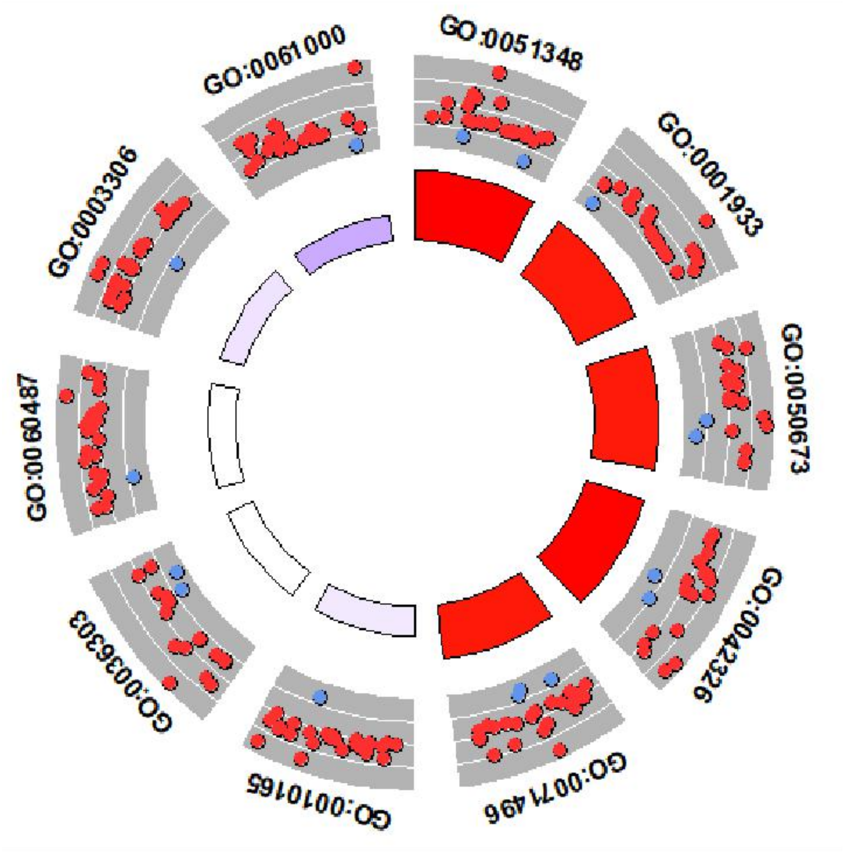

3R4F_0h

ID	Term	Count	adj_pval
GO:0051348	negative regulation of transferase activity	27	1.69e-6
GO:0042326	negative regulation of phosphorylation	34	6.13e-6
GO:0071496	cellular response to external stimulus	26	4.55e-5
GO:0050673	epithelial cell proliferation	33	3.56e-6
GO:0001933	negative regulation of protein phosphorylation	33	3.17e-6
GO:0061000	negative regulation of dendritic spine development	3	3.29e-2
GO:0003306	Wnt signaling pathway involved in heart development	3	3.29e-2
GO:0010165	response to X-ray	5	1.31e-2
GO:0036303	lymph vessel morphogenesis	4	1.45e-2
GO:0060487	lung epithelial cell differentiation	4	2.17e-2

Figure S1.

B

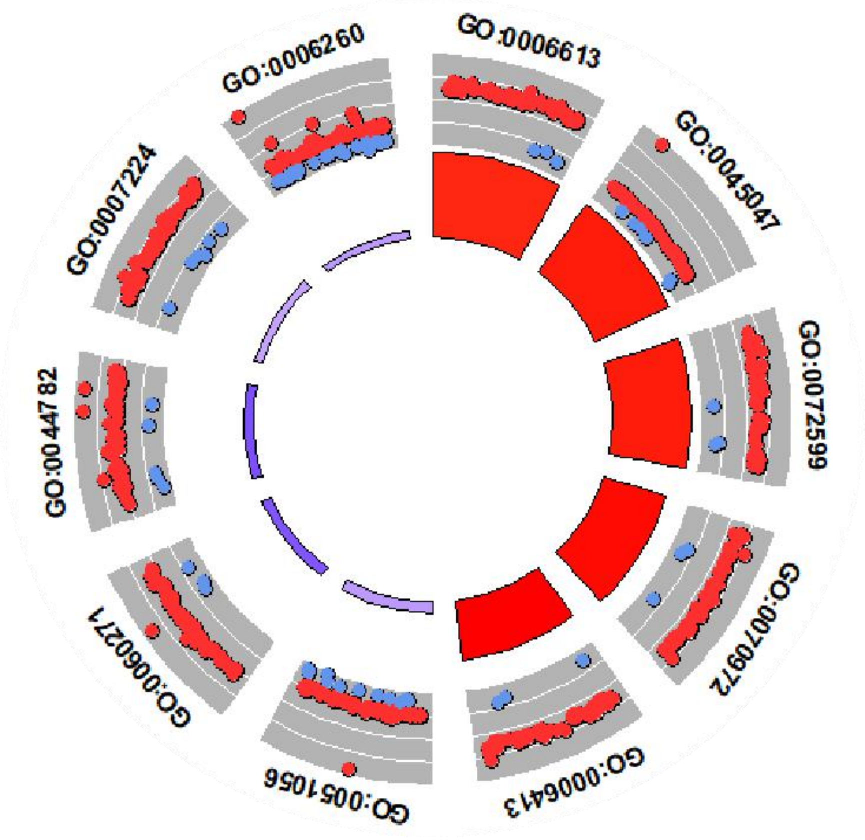

3R4F_4h

ID	Term	Count	adj_pval
GO:0006413	translational initiation	73	1.96e-18
GO:0070972	protein localization to endoplasmic reticulum	65	1.43e-20
GO:0072599	establishment of protein localization to endoplasmic reticulum	63	1.93e-24
GO:0045047	protein targeting to ER	63	2.01e-25
GO:0006613	cotranslational protein targeting to membrane	61	5.87e-26
GO:0044782	cilium organization	71	1.20e-3
GO:0060271	cilium assembly	69	9.09e-4
GO:0006260	DNA replication	51	6.24e-3
GO:0051056	regulation of small GTPase mediated signal transduction	66	4.99e-4
GO:0007224	smoothened signaling pathway	30	4.23e-3

Figure S1.

C

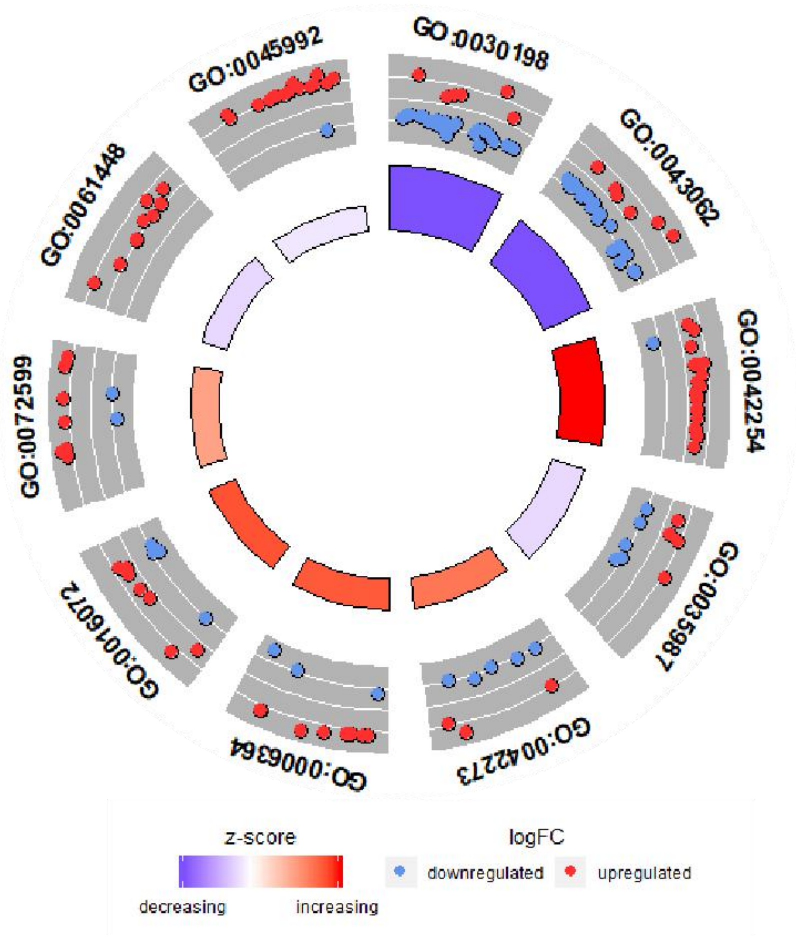

3R4F_24h

ID	Term	Count	adj_pval
GO:0042254	ribosome biogenesis	23	6.834e-4
GO:0016072	rRNA metabolic process	17	2.58e-2
GO:0006364	rRNA processing	16	1.63e-2
GO:0042273	ribosomal large subunit biogenesis	9	1.61e-2
GO:0072599	establishment of protein localization to endoplasmic reticulum	11	2.63e-2
GO:0030198	extracellular matrix organization	30	1.16e-5
GO:0043062	extracellular structure organization	30	1.30e-4
GO:0061448	connective tissue development	17	3.39-2
GO:0035987	endodermal cell differentiation	8	6.15e-3
GO:0045992	negative regulation of embryonic development	5	3.90e-2

Figure S1 GO enrichment (BP level) circle plots for the top five most significant GO categories with positive and negative z-score under 3R4F cigarette smoke treatment with three time points (A for 0h, B for 4h, C for 24h). The outer circle shows the relative fold change for each significant RNA feature compared to air contributing to the GO term, where blue dots represent down-regulated RNA features and red dots represent up-regulated features. The inner quadrants are colored based on the z-score, and its surface is a function of the enriched q-value. The larger the surface, the lower the q-value. The Z-score color scale represents the number of up-regulated genes for a given GO term minus the number of down-regulated genes divided by the square root of the total count. The related table shows the GO item ID and function, the number of RNA features that contribute to the enrichment of the GO item, and the adjusted p-value of the enrichment of the GO item.

A

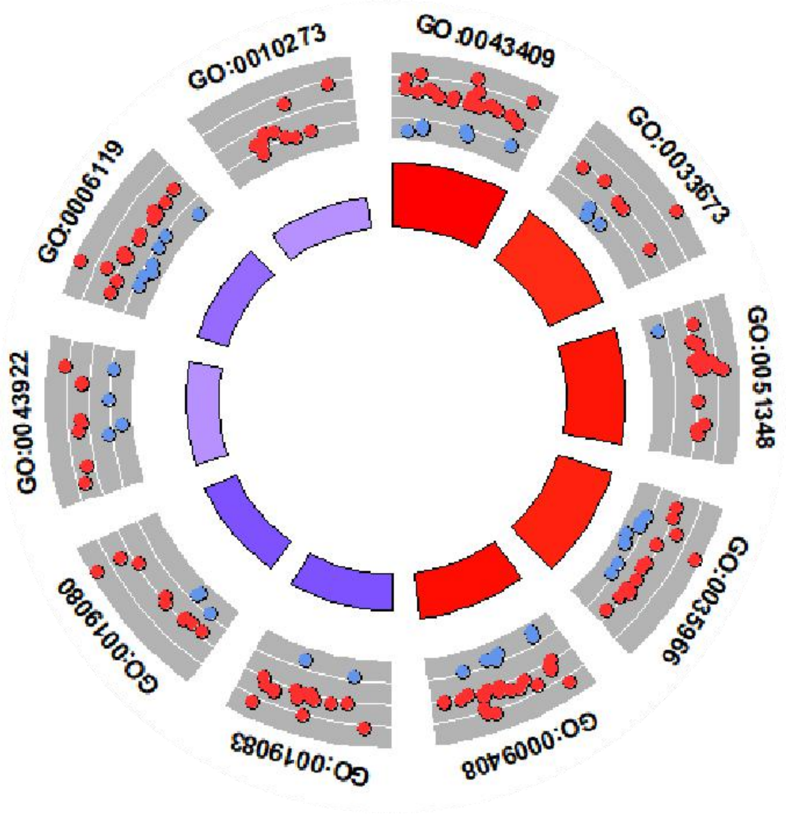

CB8_0h

ID	Term	Count	adj_pval
GO:0043409	negative regulation of MAPK cascade	17	6.78e-6
GO:0009408	response to heat	13	2.1e-3
GO:0051348	negative regulation of transferase activity	20	1.67e-4
GO:0035966	response to topologically incorrect protein	16	2.43e-4
GO:0033673	negative regulation of kinase activity	19	1.57e-4
GO:0019083	viral transcription	11	1.35e-2
GO:0019080	viral gene expression	11	2.00e-2
GO:0006119	oxidative phosphorylation	9	2.71e-2
GO:0043922	negative regulation by host of viral transcription	3	2.20e-2
GO:0010273	detoxification of copper ion	3	3.29e-2

Figure S2.

B

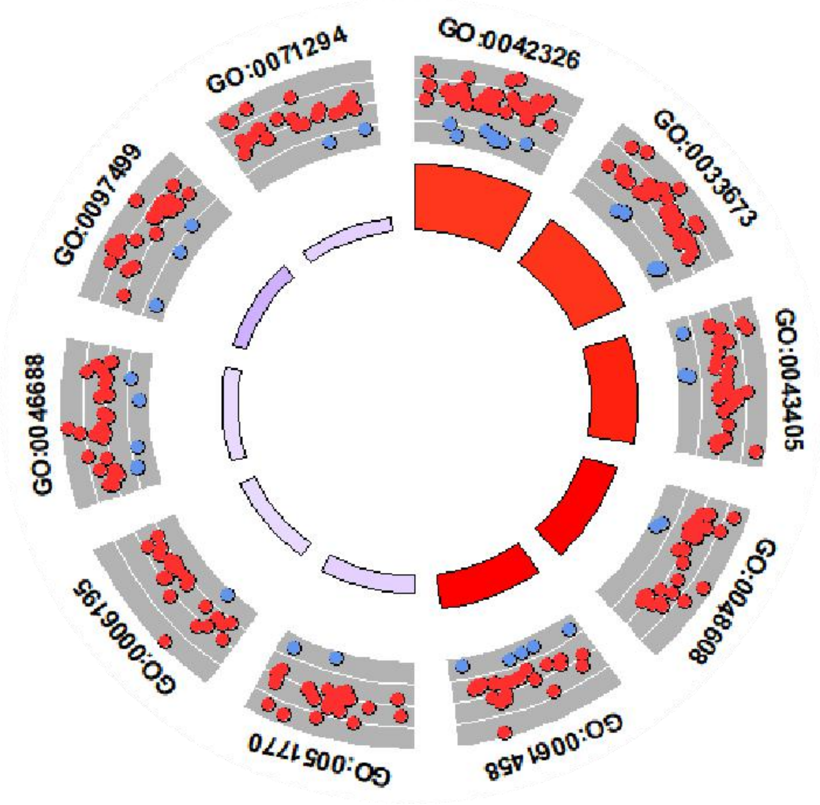

CB8_4h

ID	Term	Count	adj_pval
GO:0061458	reproductive system development	34	9.24e-05
GO:0048608	reproductive structure development	34	8.32e-05
GO:0043405	regulation of MAP kinase activity	32	6.67e-06
GO:0033673	negative regulation of kinase activity	30	4.80e-07
GO:0042326	negative regulation of phosphorylation	46	2.29e-08
GO:0097499	protein localization to non-motile cilium	3	2.55e-2
GO:0051770	positive regulation of nitric-oxide synthase biosynthetic process	4	9.35e-3
GO:0071294	cellular response to zinc ion	4	3.01e-2
GO:0006195	purine nucleotide catabolic process	6	1.76e-2
GO:0046688	response to copper ion	6	1.76e-2

Figure S2.

C

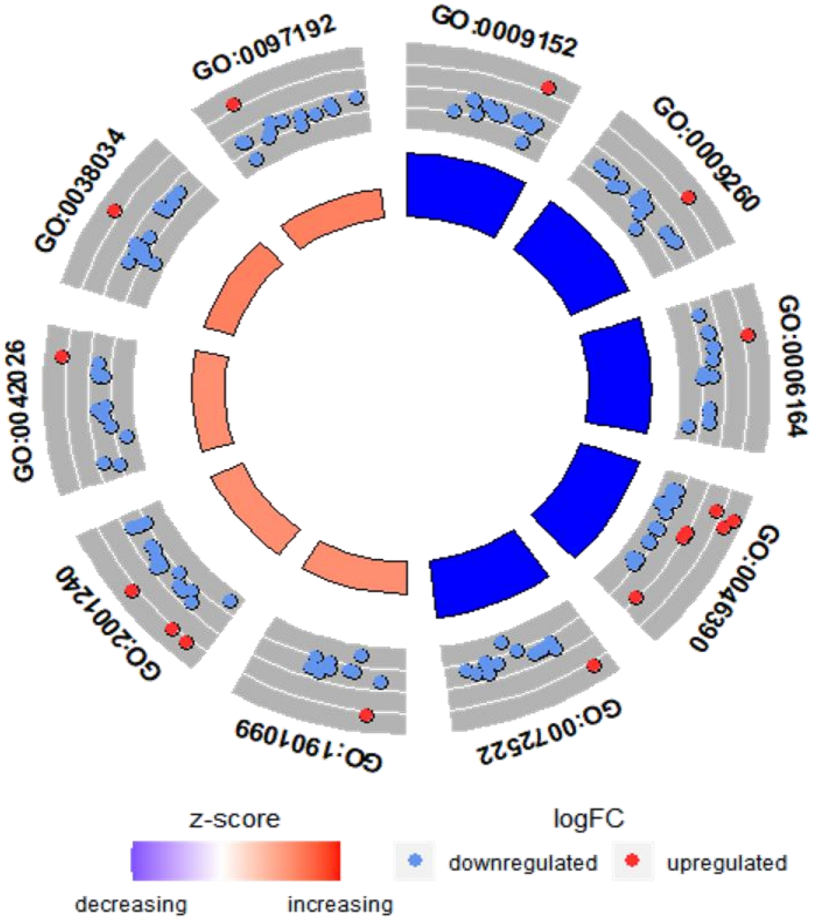

CB8_24h

ID	Term	Count	adj_pval
GO:0097192	extrinsic apoptotic signaling pathway in absence of ligand	5	4.13e-2
GO:0038034	signal transduction in absence of ligand	5	4.13e-2
GO:0042026	protein refolding	4	3.30e-2
GO:2001240	negative regulation of extrinsic apoptotic signaling pathway in absence of ligand	4	2.56e-2
GO:1901099	negative regulation of signal transduction in absence of ligand	4	2.56e-2
GO:0009152	purine ribonucleotide biosynthetic process	16	7.70e-6
GO:0009260	ribonucleotide biosynthetic process	16	1.24e-4
GO:0006164	purine nucleotide biosynthetic process	16	1.56e-4
GO:0046390	ribose phosphate biosynthetic process	16	156e-4
GO:0072522	purine-containing compound biosynthetic process	16	2.19e-4

Figure S2 GO enrichment (BP level) circle plots for the top five most significant GO categories with positive and negative z-score under CB8 cigarette smoke treatment with three time points (A for 0h, B for 4h, C for 24h).

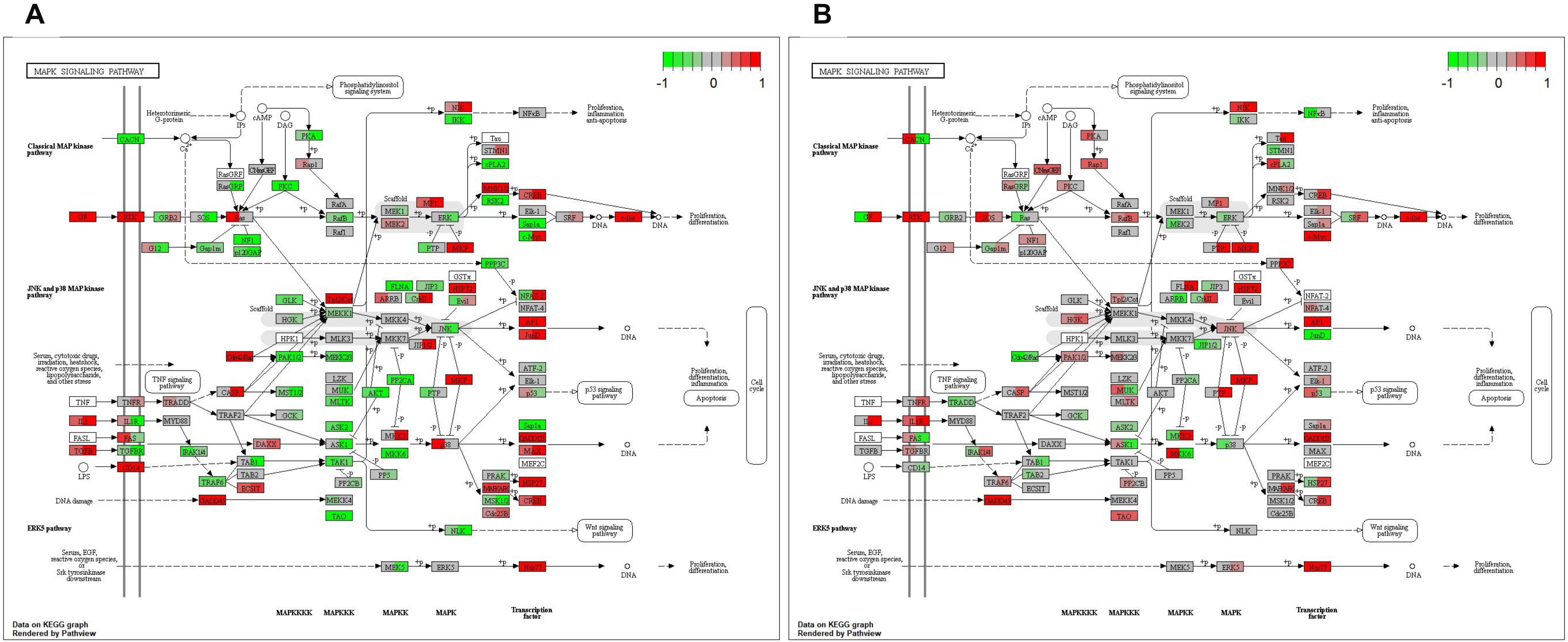

Figure S3 MAPK signaling pathway enrichment analysis under 3R4F (A) and CB8 (B) treatment.

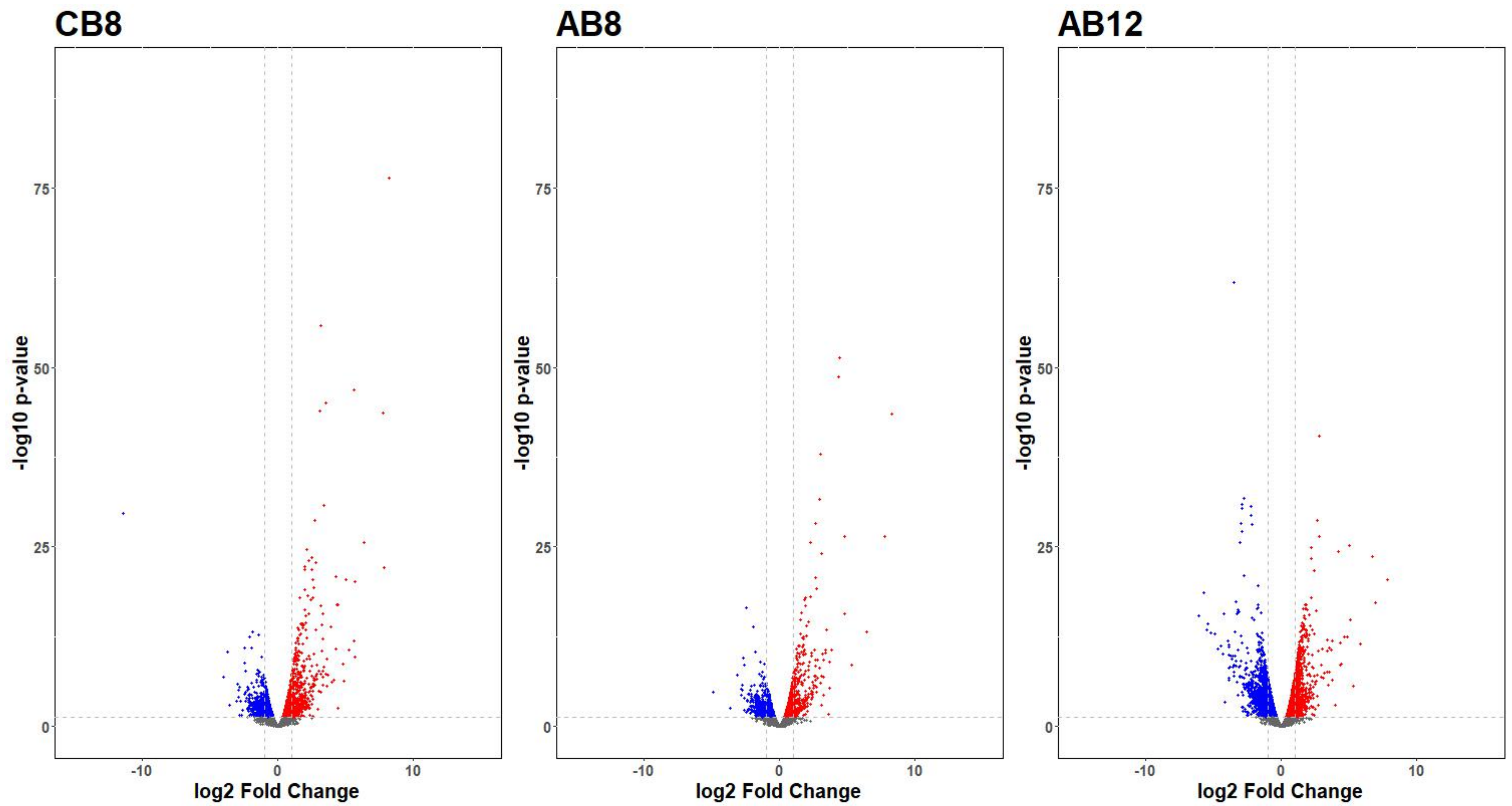

Figure S4 Volcano plots and Venn plots for the 3 comparisons (CB8, AB8 AB12). Red color indicates gene up-regulation, green color indicates gene down-regulation. Each gene block is divided into two parts, the first showing the Fold Change of the gene at 0h, and the second showing the Fold change of the gene at 4h.

A

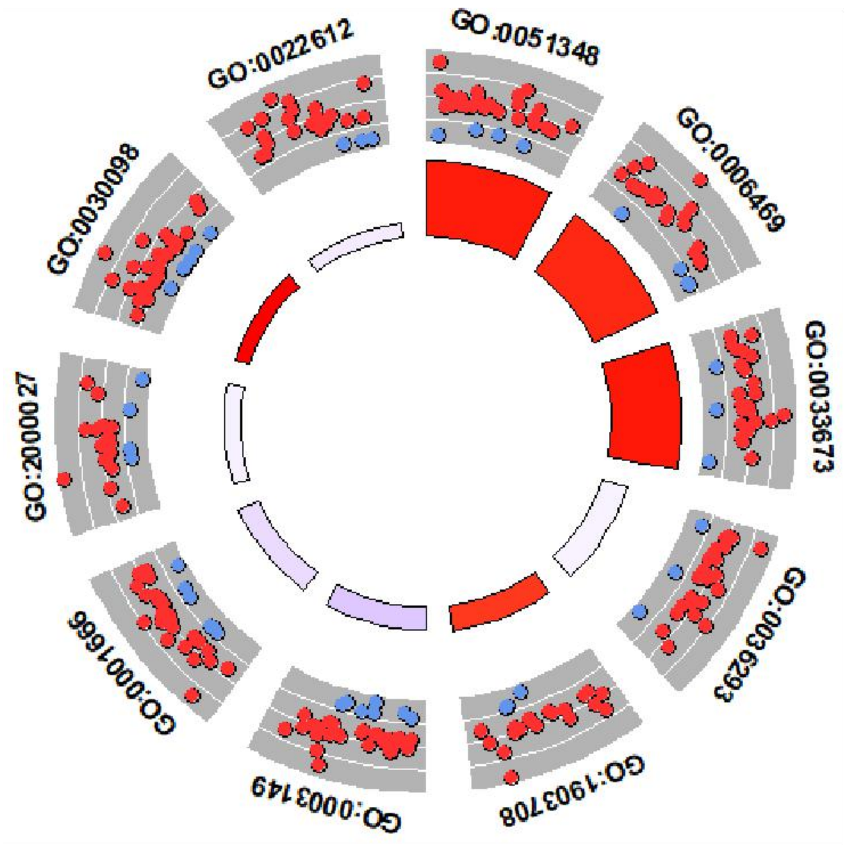

CB8

ID	Term	Count	adj_pval
GO:0030098	lymphocyte differentiation	18	1.78e-02
GO:0033673	negative regulation of kinase activity	28	4.50e-09
GO:0051348	negative regulation of transferase activity	31	1.31e-09
GO:0006469	negative regulation of protein kinase activity	27	4.15e-09
GO:1903708	positive regulation of hemopoiesis	15	1.03e-03
GO:0003149	membranous septum morphogenesis	4	1.98e-03
GO:0001666	response to hypoxia	21	2.50e-03
GO:0022612	gland morphogenesis	9	2.32e-02
GO:2000027	regulation of animal organ morphogenesis	15	1.12e-02
GO:0036293	response to decreased oxygen levels	23	7.35e-04

Figure S5.

B

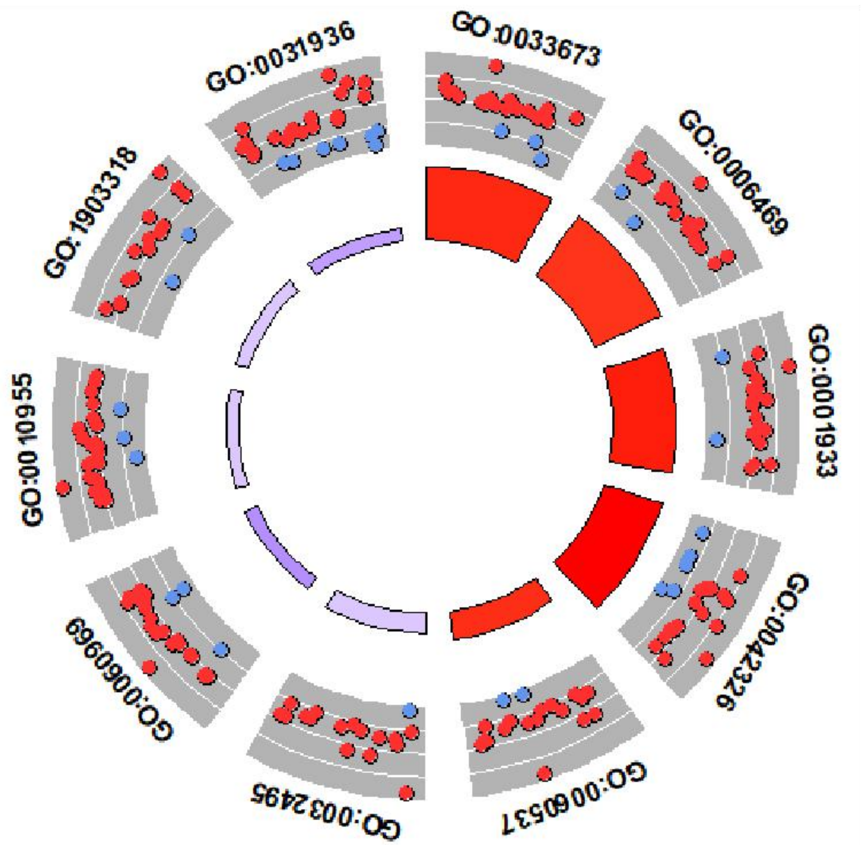

AB8

ID	Term	Count	adj_pval
GO:0042326	negative regulation of phosphorylation	28	4.71e-08
GO:0001933	negative regulation of protein phosphorylation	27	3.80e-08
GO:0033673	negative regulation of kinase activity	23	2.57e-09
GO:0060537	muscle tissue development	19	4.43e-04
GO:0006469	negative regulation of protein kinase activity	22	2.57e-09
GO:0060969	negative regulation of gene silencing	4	2.76e-2
GO:0031936	negative regulation of chromatin silencing	3	3.10e-2
GO:0032495	response to muramyl dipeptide	4	4.50e-3
GO:0010955	negative regulation of protein processing	4	3.07e-2
GO:1903318	negative regulation of protein maturation	4	3.07e-2

Figure S5.

AB12

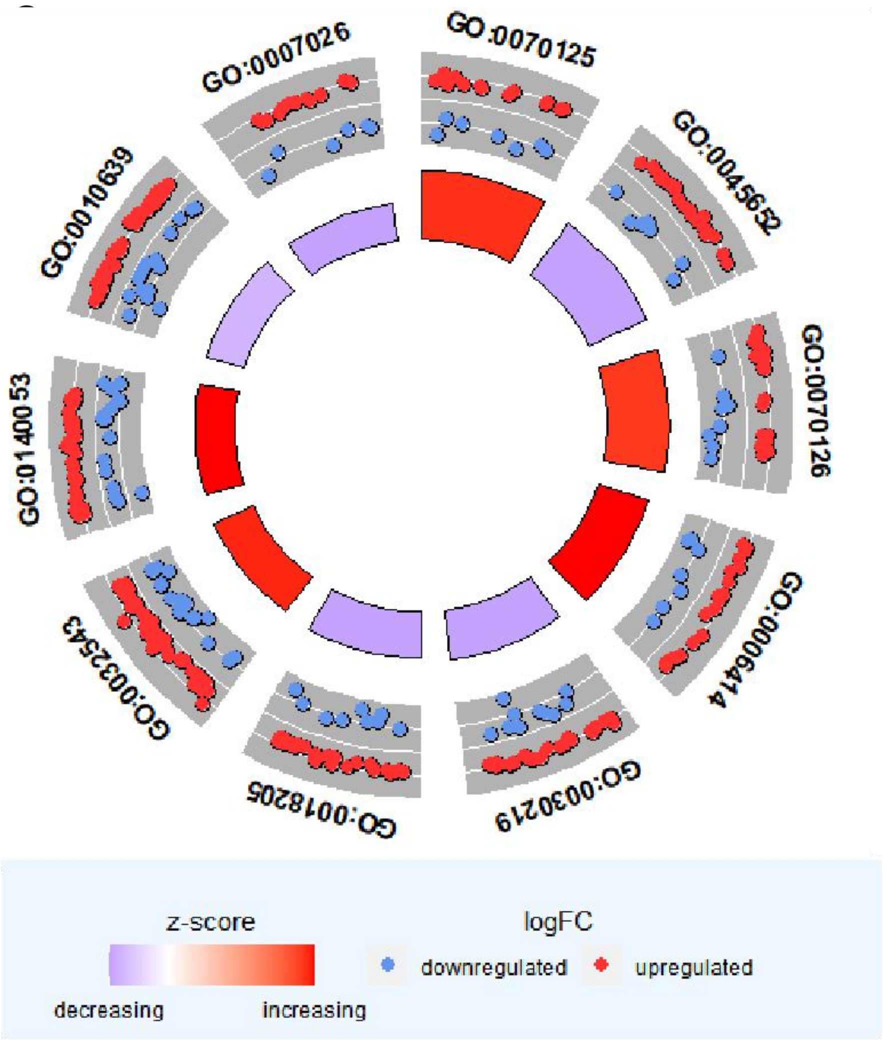

ID	Term	Count	adj_pval
GO:0140053	mitochondrial gene expression	23	2.73e-2
GO:0006414	translational elongation	23	3.54e-3
GO:0032543	mitochondrial translation	21	1.66e-2
GO:0070125	mitochondrial translational elongation	20	3.38e-4
GO:0070126	mitochondrial translational termination	19	1.03e-3
GO:0018205	peptidyl-lysine modification	47	1.01e-2
GO:0007026	negative regulation of microtubule depolymerization	7	3.73e-2
GO:0045652	regulation of megakaryocyte differentiation	18	7.76e-4
GO:0030219	megakaryocyte differentiation	18	6.45e-3
GO:0010639	negative regulation of organelle organization	44	3.28e-2

Figure S5 GO enrichment (BP level) circle plots for the top five most significant GO categories with positive and negative z-score under CB8 (A), AB8 (B) and AB12 (C) cigarette smoke treatment.
